# Field deployment of loop-mediated isothermal amplification for centralized mass-screening of asymptomatic malaria in Zanzibar: a pre-elimination setting

**DOI:** 10.1186/s12936-015-0731-2

**Published:** 2015-05-17

**Authors:** Ulrika Morris, Mwinyi Khamis, Berit Aydin-Schmidt, Ali K Abass, Mwinyi I Msellem, Majda H Nassor, Iveth J González, Andreas Mårtensson, Abdullah S Ali, Anders Björkman, Jackie Cook

**Affiliations:** Malaria Research, Department of Microbiology, Tumor and Cell Biology, Karolinska Institutet, Nobelsväg 16, 171 77 Stockholm, Sweden; Zanzibar Malaria Elimination Programme (ZAMEP), Ministry of Health, Zanzibar, Tanzania; Foundation for Innovative New Diagnostics (FIND), Geneva, Switzerland; Department of Public Health Sciences, Karolinska Institutet, Stockholm, Sweden; Centre for Clinical Research Sörmland, Uppsala University, Uppsala, Sweden; London School of Hygiene and Tropical Medicine, London, UK

**Keywords:** *Plasmodium*, Malaria, Low-density, Asymptomatic, Loop-mediated isothermal amplification, Mass screening, DNA contamination

## Abstract

**Background:**

Molecular tools for detection of low-density asymptomatic *Plasmodium* infections are needed in malaria elimination efforts. This study reports results from the hitherto largest implementation of loop-mediated isothermal amplification (LAMP) for centralized mass screening of asymptomatic malaria in Zanzibar.

**Methods:**

Healthy individuals present and willing to participate in randomly selected households in 60 villages throughout Zanzibar were screened for malaria by rapid diagnostic tests (RDT). In 50 % of the study households, participants were asked to provide 60 μL of finger-prick blood for additional LAMP screening. LAMP was conducted in two centralized laboratories in Zanzibar, by trained technicians with limited or no previous experience of molecular methods. The LAMP assay was performed with Loopamp^TM^ MALARIA Pan/Pf Detection Kit (Eiken Chemical Company, Japan). Samples positive for *Plasmodium* genus (Pan)-LAMP were re-tested using *Plasmodium falciparum*-specific LAMP kits.

**Results:**

Paired RDT and LAMP samples were available from 3983 individuals. The prevalence of asymptomatic malaria was 0.5 % (CI 95 % 0.1-0.8) and 1.6 % (CI 95 % 1.1-2.2) by RDT and Pan-LAMP, respectively. LAMP detected 3.4 (CI 95 % 2.2-5.2) times more *Plasmodium* positive samples than RDT. DNA contamination was experienced, but solved by repetitive decontamination of all equipment and reagents.

**Conclusions:**

LAMP is a simple and sensitive molecular tool, and has potential in active surveillance and mass-screening programmes for detection of low-density asymptomatic malaria in pre-elimination settings. However, in order to deploy LAMP more effectively in field settings, protocols may need to be adapted for processing larger numbers of samples. A higher throughput, affordable closed system would be ideal to avoid contamination.

**Electronic supplementary material:**

The online version of this article (doi:10.1186/s12936-015-0731-2) contains supplementary material, which is available to authorized users.

## Background

Asymptomatic *Plasmodium* infections are an important reservoir for continued malaria transmission that needs to be addressed in the context of malaria elimination [1]. Detection of asymptomatic infections, which are often sub-patent, i.e., fall beneath the threshold of detection of both microscopy and rapid diagnostic tests (RDT), requires highly sensitive molecular tools. The use of polymerase chain reaction (PCR)-based assays in field settings is, however, limited due to the need for a cold chain, specialized equipment and know-how [2]. Loop-mediated isothermal amplification (LAMP) offers several advantages over PCR in field settings. LAMP requires minimal equipment, has short time-to-result (30 min-1 h), with an analytical sensitivity similar to PCR, and results that can be read by eye using UV fluorescence [3–5].

The Loopamp™ MALARIA Pan/Pf Detection Kit (Eiken Chemical Company, Japan) has been developed as a field-friendly kit, comprising strips of reaction tubes containing vacuum-dried and temperature-stable reaction components for either *Plasmodium* genus (Pan)-specific or *Plasmodium falciparum*-specific malaria detection. The kit has been evaluated both in laboratory and field settings [6–8], and was piloted on a small scale in Zanzibar as a health facility-based, point-of-care, diagnostic tool for mass screening and treatment in 2013 [9].

This study reports results from the hitherto largest implementation of LAMP in the field, for scaled-up, centralized mass screening of asymptomatic malaria in Zanzibar, a pre-elimination setting.

## Methods

### Study sites and study design

Zanzibar, located 35 km off the coast of mainland Tanzania, consists of two main islands, Unguja and Pemba, with respective populations of approximately 900,000 and 400,000. This study was performed as part of a larger knowledge, attitude, practice, and behaviour (KAPB) malaria survey, conducted in Zanzibar April-May 2014. Household visits were carried out in 60 villages in ten districts (six in Unguja and four in Pemba) covering the whole of Zanzibar. A proportional number of households were sampled from each village to reach a sample size of 2162 households, powered for the KAPB study. Healthy individuals present and willing to participate in the randomly selected households were screened for malaria by RDT. In 50 % of study households (in even house numbers), participants were asked to provide 60 μL of finger-prick blood for additional LAMP screening. Nexus seven tablet computers were used to conduct questionnaires as part of the KAPB survey. All participants or guardians provided written informed consent prior to blood sampling. Ethical approvals were obtained from the ethical committees in Zanzibar (ZAMREC/0002/FEBRUARY/014) and the Regional Ethics Committee in Stockholm (2009/387-31).

### Training of field enumerators and sample collection

Household visits were conducted by 40 field enumerators in teams of two, together with four field supervisors with prior experience of similar studies. All enumerators attended five days of training for RDT performance, blood sample collection for LAMP, and use of tablet computers. There were 14 teams in Unguja and six teams in Pemba, and each team visited six or seven households per day. RDT screening was conducted with either SD-Bioline Malaria Ag P.f/Pan® (Standard Diagnostic, Inc, USA) (used for >90 % of screening) or *First Response*® Malaria Ag Combo (pLDH/HRP2) (Premier Medical Corporation Limited India). Results were recorded on the tablet computer during household visits, and RDT positive individuals were referred to the closest health facility for treatment and registration in the Zanzibar malaria surveillance system. In 50 % of study households, 60 μL of finger-prick blood was collected using a plastic capillary tube (Dropstir, Medical Precision Plastics, USA), dispensed into a 1.5-ml pre-labelled sample collection tube containing 60 μL of pre-aliquoted DNA extraction buffer (400 nM NaCl, 40 mM Tris pH 6.5, 0.45 SDS), and mixed by flicking. Blood samples were collected in microtube storage racks with lids and transported at the end of each day to two centralized laboratories, one on each island, where they were stored at 4 °C overnight.

### Training of laboratory technicians

Four technicians, two for each laboratory, with limited or no experience of LAMP were trained over three-and-a-half days. Training included a theoretical introduction to LAMP and the LAMP protocol, hands-on practical sessions with malaria positive blood samples diluted to different known concentrations, how to record results on tablet computers, and a half-day field trial with samples collected the same day by the field enumerators.

### Screening by LAMP in centralized laboratories

LAMP procedures were similar to the pilot study [9], with some modifications for scale-up of sample sizes. One centrifuge, three heat-blocks (1.5-ml block at 95 °C, 0.2-ml block at 65 °C and a 0.2-ml block at 95 °C) and a UV lamp were required in each laboratory. All samples collected in Pemba and half of the samples collected in Unguja (see below) were processed within 24 h of sampling. To reduce the risk of mix-up of samples and contamination, sets of pre-labelled sample collection tubes (containing 60 μL of aliquoted DNA extraction buffer) and pre-labelled DNA dilution tubes (containing 300 μL of aliquoted sterile water) were prepared prior to the start of the study. DNA extraction and the LAMP assays were performed in separate areas to avoid contamination. DNA was extracted by the boil and spin method [10] and 26 μL of the supernatant was transferred to the DNA dilution tubes. The LAMP assay was performed with Loopamp™ MALARIA Pan/Pf Detection Kit (Eiken Chemical Company) as per protocol [10]. Samples positive for Pan-LAMP were retested using *P. falciparum*-LAMP specific kits. LAMP positive individuals (who were not positive by RDT) were visited by malaria surveillance officers and provided treatment within 48 h of sampling where possible.

### Freezing of samples

Due to a delay in the shipment of LAMP kits, half of the LAMP samples collected in Unguja (*N* = 1414) were stored at −20 °C after DNA extraction and dilution, until the remaining reaction tubes arrived five weeks later. Dilution tubes from LAMP-positive samples in Unguja were also stored at −20 °C, for quality control of frozen DNA.

### Statistical analysis

Results are reported from individuals for which both RDT and LAMP were conducted (i.e., where paired data are available). Statistical analyses were conducted using Stata/SE 12.1 (StataCorp LP, Texas, USA). The survey design was taken into consideration when calculating 95 % confidence intervals (CI 95 %) for prevalence estimations, using the survey [svy] command in Stata accounting for household and village sampling/stratification. The sensitivity and specificity of RDT was calculated using LAMP as the gold standard. McNemar’s test was used to compare the methods. Statistical significance was determined as *p* <0.05.

## Results

### Study population

**Fig. 1:**
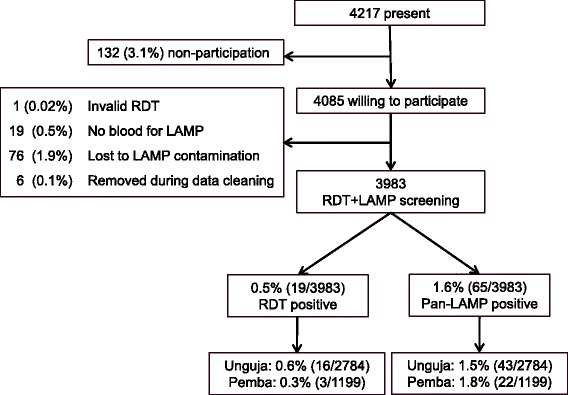
Flow chart of study

Participation was high; informed consent was given by 96.9 % of those present at the time of the survey (Fig. 1). Both RDT and LAMP results were available for 3983/4085 (97.5 %) of the individuals willing to participate. The remaining 102 (2.5 %) were excluded from further analysis. The study population consisted of all ages (median: 18 years, range 0–98), with a higher proportion of females (59.0 %). Sample collection was conducted during a total of 19 days with an average of 220 samples processed per day in the two laboratories combined.

### Prevalence of malaria by RDT and LAMP

The prevalence of asymptomatic malaria was 0.5 % (CI 95 % 0.1-0.8) and 1.6 % (CI 95 % 1.1-2.2) by RDT and Pan-LAMP, respectively (Table 1). Pan-LAMP detected 3.4 (CI 95 % 2.2-5.2) times more *Plasmodium* positive samples than RDT. Out of the Pan-LAMP positive samples 64.6 % (42/65) were also positive by *P. falciparum*-LAMP. RDT had a sensitivity of 24.6 % (14.7-36.9) and specificity of 99.9 % (99.7-100.0) when compared to Pan-LAMP. Comparison by McNemar’s test showed a significant difference between the two methods (*p* <0.001).

**Table 1:** Prevalence of malaria detected by RDT and LAMP

	RDT	LAMP
Overall prevalence (%; CI 95 %^a^)	0.5; 0.1-0.8	1.6; 1.1-2.2
	19/3983	65/3983
Relative proportion positive in:		
Only Pan^b^ (%; CI 95 %)	5.3; 0.0-16.4	35.4; 23.4-47.4
	1/19	23/65
Pan + *P. falciparum* ^c^ (%; CI 95 %)	31.6; 8.5-54.6	64.6; 52.6-76.6
	6/19	42/65
Only *P. falciparum* ^d^ (%; CI 95 %)	63.2; 39.2-87.1	ND^e^
	12/19	

Both RDT brands used for malaria screening are two-band RDTs detecting *P. falciparum* HRP2 and Pan-*Plasmodium* LDH simultaneously, although with different detection limits (50–100 parasites/μL for *P. falciparum* HRP2 and 200–500 parasites/μL for Pan-*Plasmodium* LDH). In contrast, only the Pan-LAMP positive samples were assessed for *P. falciparum* during the LAMP screening, with a detection limit of 2–5 parasites/μL for both Pan-*Plasmodium* and *P. falciparum*

^a^Confidence intervals for prevalences were calculated using the survey [svy] command in Stata, accounting for household and village sampling/stratification

^b^Positive for *Plasmodium* genus only

^c^Positive for *Plasmodium* and *P. falciparum*

^d^Positive for *P. falciparum* only

^e^ND = not determined

### Discrepancies in LAMP after freezing of samples

DNA extracted from half of the samples (*N* = 1414) in Unguja was stored at −20 °C prior to LAMP testing due to a delay in the shipment of LAMP kits. Among these samples, 32 (2.3 %) were positive by Pan-LAMP, out of which 12 was also positive by RDT. However, amongst the frozen samples there were also three RDT positive samples that were found negative by Pan-LAMP. These three samples were positive for *P. falciparum* HRP2 only, Pan-*Plasmodium* LDH only, and both *P. falciparum* HRP2 and Pan-*Plasmodium* LDH, respectively. Among the samples from Unguja that were screened before freezing (*N* = 1370), 11 (0.8 %) were positive by Pan-LAMP, out of which one was also positive by RDT. The 11 Pan-LAMP positive samples were stored at −20 °C, as a quality control of freezing DNA, however only 7/11 (63.6 %) were positive when re-tested after thawing.

### LAMP-amplified DNA contamination

During the study DNA contamination of LAMP arose in the central laboratory in Pemba [see Additional file 1 for flow chart of events]. The contamination resulted from using a heat block with a heated pressurized lid during the 95 °C enzyme inactivation stage, and not allowing the samples to cool to room temperature before removing the strips for recording of results. ‘Fizzing’ was observed around the lid of the LAMP strips resulting in leakage of LAMP-amplified DNA. All equipment and reagents were subjected to repetitive decontamination with 5 % sodium hypochlorite over three days, and moved away from the epicentre of the contamination to a laboratory space available in another building. The final enzyme inactivation step of the protocol [10] was removed as this was thought to be the source of contamination; instead results were read and recorded immediately after the amplification reaction. A negative control was included in each strip of eight reaction tubes, and any Pan-LAMP positive samples were repeated and only recorded as positive if positive in both runs. During the first few days following the contamination there were some samples that were considered false positive, but the numbers declined and reached zero within one week after the contamination.

## Discussion

This is the hitherto largest reported implementation of LAMP for detection of asymptomatic malaria in a field setting. In order to scale-up the breadth of sampling, LAMP testing was centralized in two laboratories, meaning samples could be collected from all over the islands with fewer resources. The time-to-result was approximately 24 h, compared with three hours in the pilot study where LAMP was used as a health facility-based, point-of-care, diagnostic tool for mass screening and treatment [9].

The results confirm the improved sensitivity of LAMP over RDT, as has been shown previously [3, 9]. The MALARIA Pan/Pf Detection Kit has a detection limit of 2–5 parasites/μL [3, 6], for both Pan-*Plasmodium* and *P. falciparum*. This is comparable to PCR, and substantially lower than the detection limits of *P. falciparum*-specific HRP2 (50–100 parasites/μL) and Pan-*Plasmodium* LDH (200–500 parasites/μL) in combo RDTs. The proportion of samples detected only by Pan-LAMP (35.4 %) suggests the presence of species other than *P. falciparum*. Similarly, other studies in Zanzibar have shown that up to 40 % of PCR-detectable malaria infections contained non-falciparum species [11, 12]. Non-falciparum infections tend to be of lower parasite densities than *P. falciparum* infections [13], emphasizing the need for more sensitive species-specific methods for non-falciparum *Plasmodium* detection. The sensitivity (83.8 %) and specificity (99.7 %) of Pan-LAMP, calculated using PCR as the reference standard, was high in the pilot study conducted in Zanzibar [9]. This is similar to previously reported sensitivities and specificities [6–8, 14, 15] and, together with the results of this study, suggests malaria LAMP is a useful molecular tool sensitive enough for detection of low-density asymptomatic malaria infections in field settings.

Importantly, some discrepancies were shown amongst samples screened following freezing of diluted DNA. RDT false positivity due to recently cleared infections has been well documented when detecting *P. falciparum* HRP2 [16], although none of the three study participants who were RDT positive/LAMP negative reported receiving malaria treatment within the previous two weeks, and two of the RDTs were positive for Pan-*Plasmodium* LDH suggesting ongoing infections. The lack of reproducibility of results following freezing of samples suggests that DNA extracted by simple methods such as boil and spin may not be suitable for long-term storage and should be amplified by LAMP within a short period of time [17]. Alternatively, low reproducibility of PCR for detection of low-density infections has been reported [18] and parasite densities close to the LAMP detection limit could also explain the lack of reproducibility.

The potential risk of contamination with LAMP is large, due to the high efficiency of the reaction, although the risk is reduced when using a closed system [3, 19]. The MALARIA Pan/Pf Detection Kit is manufactured with tubes that cannot be re-opened once closed, in order to avoid contamination with amplified DNA. However, as demonstrated in this study, the exposure of such tubes to high temperatures, as during enzyme inactivation, results in softening of the plastic and leakage of the contents. While removing the inactivation step solved this problem in this case, contaminations have been experienced in other research settings [8, 20] and these issues are important to report. Although MALARIA Pan/Pf Detection Kit is a field-friendly option, three days’ training is not sufficient for dealing with such events. Successful decontamination requires a larger understanding of molecular techniques and rigorous repetitive methods to ensure that the area is free of contamination.

Standard malaria diagnostic tools including microscopy and RDT are not sensitive enough to detect low-density asymptomatic infections [12]. Nucleic acid amplification-based methods provide the, to date, most sensitive and accurate tools to detect and identify pathogens [21]. Recently published, highly sensitive quantitative PCR methods state detection limits as low as 0.02 and 0.03 parasites/μL blood [22, 23]. However, these methods lack the field applicability that LAMP offers. Furthermore, the cost of LAMP is estimated to be a tenth of that of conventional PCR [15], although the cost of the field friendly kit is still at 5.3 US$ per reaction i.e., considerably more expensive than RDTs [3].

The high cost and risk of contamination may yet limit the implementation LAMP at a point-of-care level, but LAMP will be valuable for research purposes and for evaluating malaria elimination efforts. LAMP may, for example, be useful in mass/focal screening and treatment (MSAT/FSAT) programmes, for which the deployment of RDTs, perhaps due to their low sensitivity, has had varying results [11, 24]. In any case it will be important to evaluate the impact and cost effectiveness of deploying LAMP, in comparison to the deployment of standard diagnostic tools as well as in comparison to alternative molecular methods.

## Conclusions

LAMP is a simple and sensitive molecular tool, and has potential in active surveillance and mass-screening programmes for detection of low-density asymptomatic malaria in pre-elimination settings. However, in order to deploy LAMP more effectively in field settings, protocols may need to be adapted for processing larger numbers of samples. A higher throughput, affordable closed system would be ideal to avoid contamination.

## Additional file

Additional file 1:
**Flow chart of events that took place during LAMP DNA contamination and decontamination.** A figure showing the flow of events that took place during a DNA contamination when screening for asymptomatic malaria by LAMP in Zanzibar The flow chart shows how the DNA contamination was resolved by repetitive decontamination of all equipment with 5 % sodium hypochlorite and moving away from the site of contamination.
