# Supplementary material for: Field deployment of loop-mediated isothermal amplification for centralized mass-screening of asymptomatic malaria in Zanzibar: a pre-elimination setting
Source: Malar J. 2015 May 17;14:205. doi: 10.1186/s12936-015-0731-2 (PMC4440539; doi:10.1186/s12936-015-0731-2)
Supplement: Additional file 1: — Flow chart of events that took place during LAMP DNA contamination and decontamination. A figure showing the flow of events that took place during a DNA contamination when screening for asymptomatic malaria by LAMP in Zanzibar The flow chart shows how the DNA contamination was resolved by repetitive decontamination of all equipment with 5 % sodium hypochlorite and moving away from the site of contamination. [file 12936_2015_731_MOESM1_ESM.pdf]

Before contamination

In each run: 46 samples with 1 positive + 1 negative control. Heat inactivation at 95 °C for 2 min (as per protocol), in a heat block with a heated pressurized lid (ThermoQ metal bath, Bioer, Hangzhou, China)

DNA-contamination

50-100% of samples and the negative control were LAMP positive, even after cleaning lab surfaces and equipment

De-contamination process

1. Removed all necessary lab equipment and consumables from the contaminated area
2. Cleaned <u>all</u> equipment and consumables three times with 5% sodium hypochlorite (household bleach)
3. Changed clothes. Clothes, hands and hair can carry amplified DNA and will continue to contaminate the surroundings
4. Moved all equipment to an intermediate space for quality control
5. Ran 3 x 8 reaction tubes with negative controls and one positive control (It was during this step, under close observation, that we observed fizzing around the lid of the reaction tubes after heat inactivation)
6. Heat inactivation stage was removed from the protocol
7. Step 5 was repeated until all negative controls were truly negative. All equipment was cleaned in between each repeat (this took several repeats)
8. Moved to a non-contaminated lab space in another building
9. Again, cleaned all equipment, changed clothes, lab coats, gloves etc. Re-did quality control with 3x8 reaction tubes until all controls were negative

Post-contamination

Heat inactivation stage removed from protocol, results read immediately after 40 min at 65 °C. Negative control included in each strip of 8 reaction tubes (positive control only included in 1/6 strips). Any positive samples were repeated, and only recorded as positive if positive in both runs

A few samples were considered false positive during the first week after the contamination, but this number eventually reached zero
